# Supplementary material for: LocusMasterTE: integrating long-read RNA sequencing improves locus-specific quantification of transposable element expression
Source: Genome Biol. 2025 Mar 26;26:72. doi: 10.1186/s13059-025-03522-9 (PMC11948968; doi:10.1186/s13059-025-03522-9)
Supplement: Supplementary file 1 — Additional file 1. Supplementary figures of LocusMasterTE: integrating long-read RNA sequencing improves locus-specific quantification of transposable element expression file contains Fig. S1 – S8. [file 13059_2025_3522_MOESM1_ESM.pdf]

S1

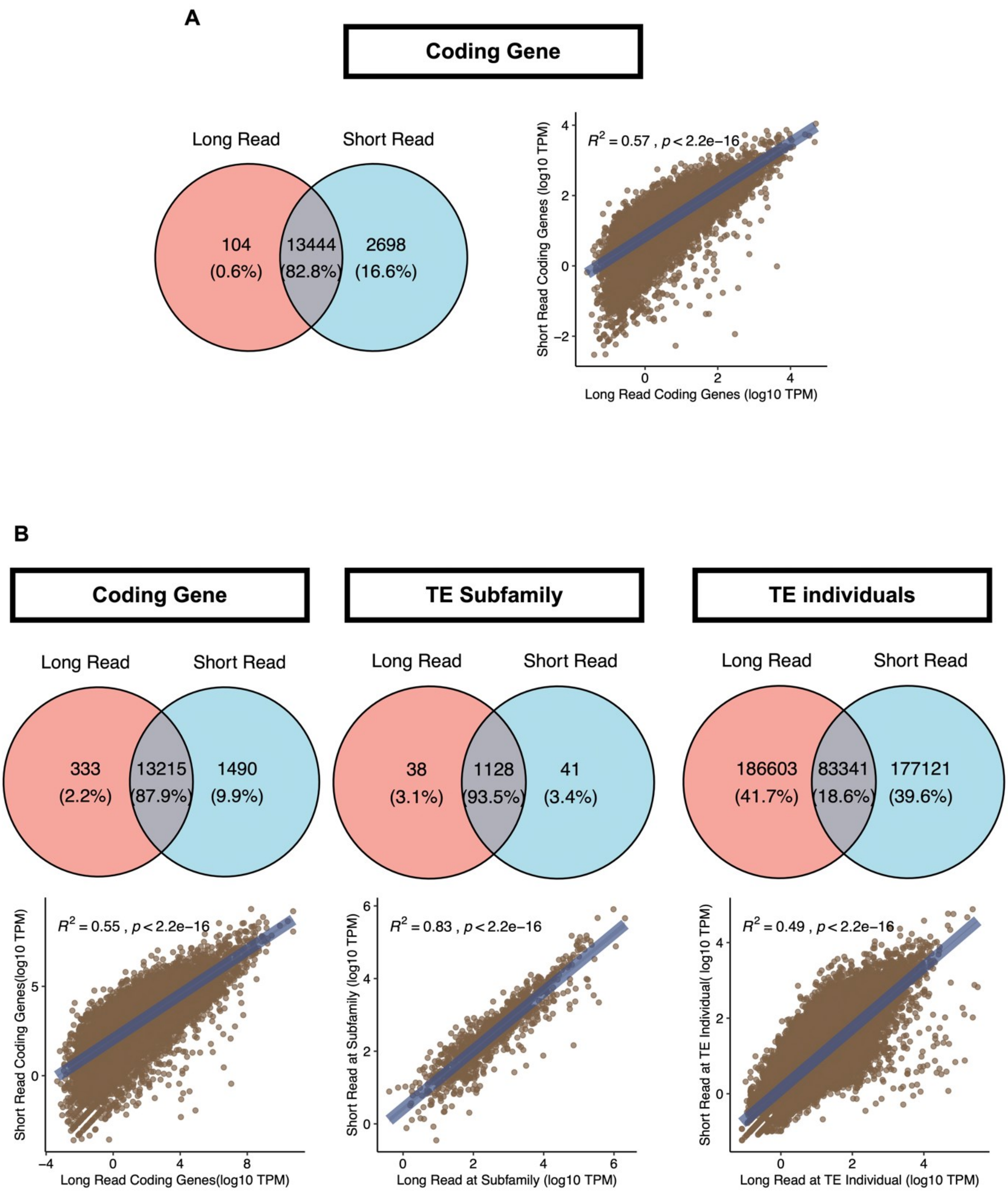

Fig S1: Comparison between short-read and long-read

(A) Comparison of coding genes captured by short-read and long-read RNA-seq. Venn diagram showing the number of captured genes in two RNA-seq techniques. Spearman correlation plot between TPM count for short-read and long-reads in the coding genes domain. (B) Comparison between downsampled short-read and long-read RNA-seq. The short-read sample is downsampled to match the number of bases mapped between short-read and long-read. Venn diagrams for downsampled short-read and long-read of TEs in subfamily and individual levels. Spearman correlation plots between TPM count for downsampled short-read and long-reads in the same domains.

S2

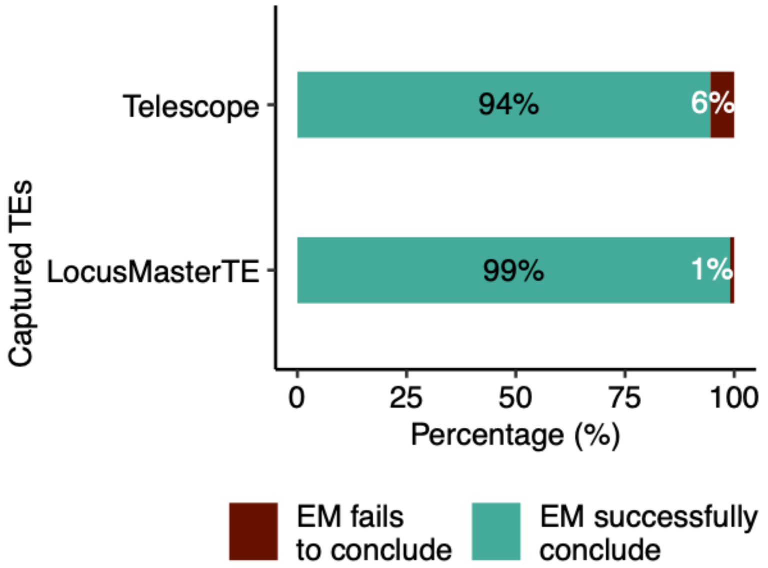

Fig S2: A comparison between Telescope and LocusMasterTE in the proportion of cases where EM successfully identifies the best hit

Bar plot showing the proportion of cases that EM fails to determine a single genomic position that multi-mapped reads mapped to. Aided by long-read, LocusMasterTE reduced those cases to 1%.

S3

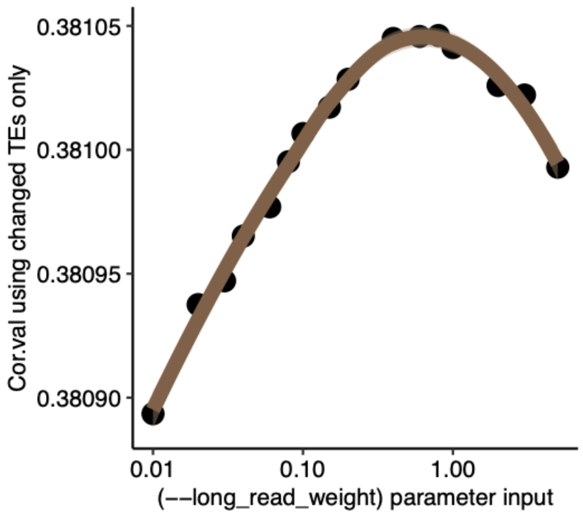

Fig S3: Impact of (--*long_read_weight*) parameter in LocusMasterTE
TE expressions of the inhouse HCT116 sample with K562 long-read with varied (--*long_read_weight*) values and a series of correlation tests were conducted with the inhouse HCT116 long-read sample. Log TPM values were used for all TE expressions. For clear demonstration, only TEs with expression changed with (--*long_read_weight*) values are considered.

S4

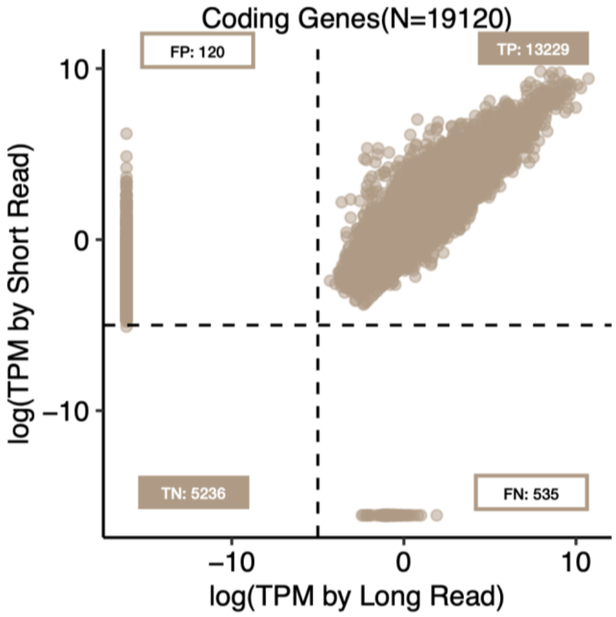

Fig S4: Simulated short-read for coding genes

Correlation plot for 19120 coding genes between simulated short-read and long-read. This analysis is performed to check the success of the simulation. As expected, most of the coding genes are correctly simulated.

S5

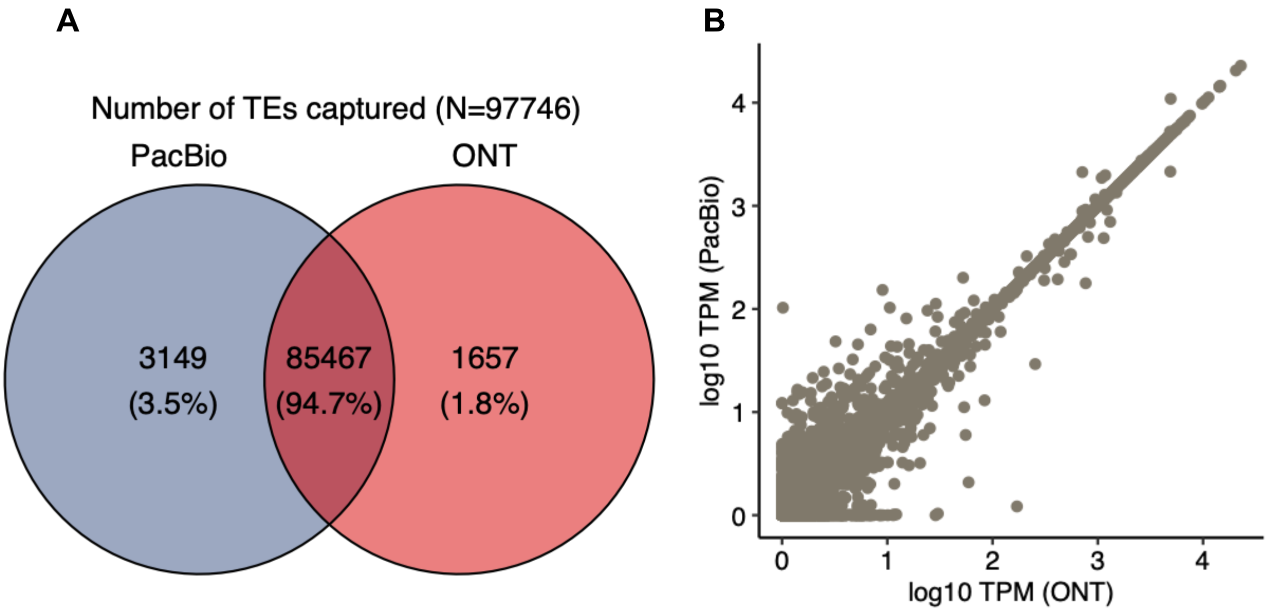

Fig S5: Quantified TE expression between Nanopore vs PacBio on simulated short-read
(A) Venn diagram showing the number of TE individuals captured by LocusMasterTE using PacBio vs. ONT. (B) Correlation plot between quantified TEs by LocusMasterTE using PacBio as long-read input vs ONT as long-read input.

S6

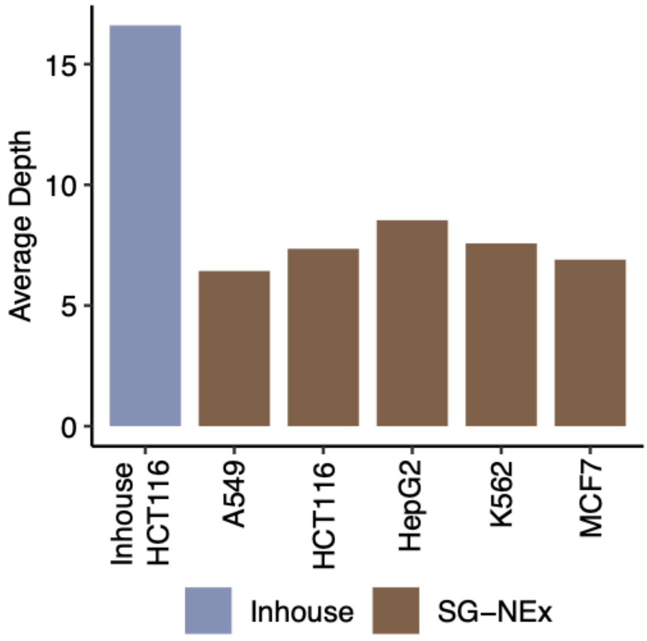

Fig S6: Average depth across all 6 samples
Bar plot showing average read depth across all 6 samples.

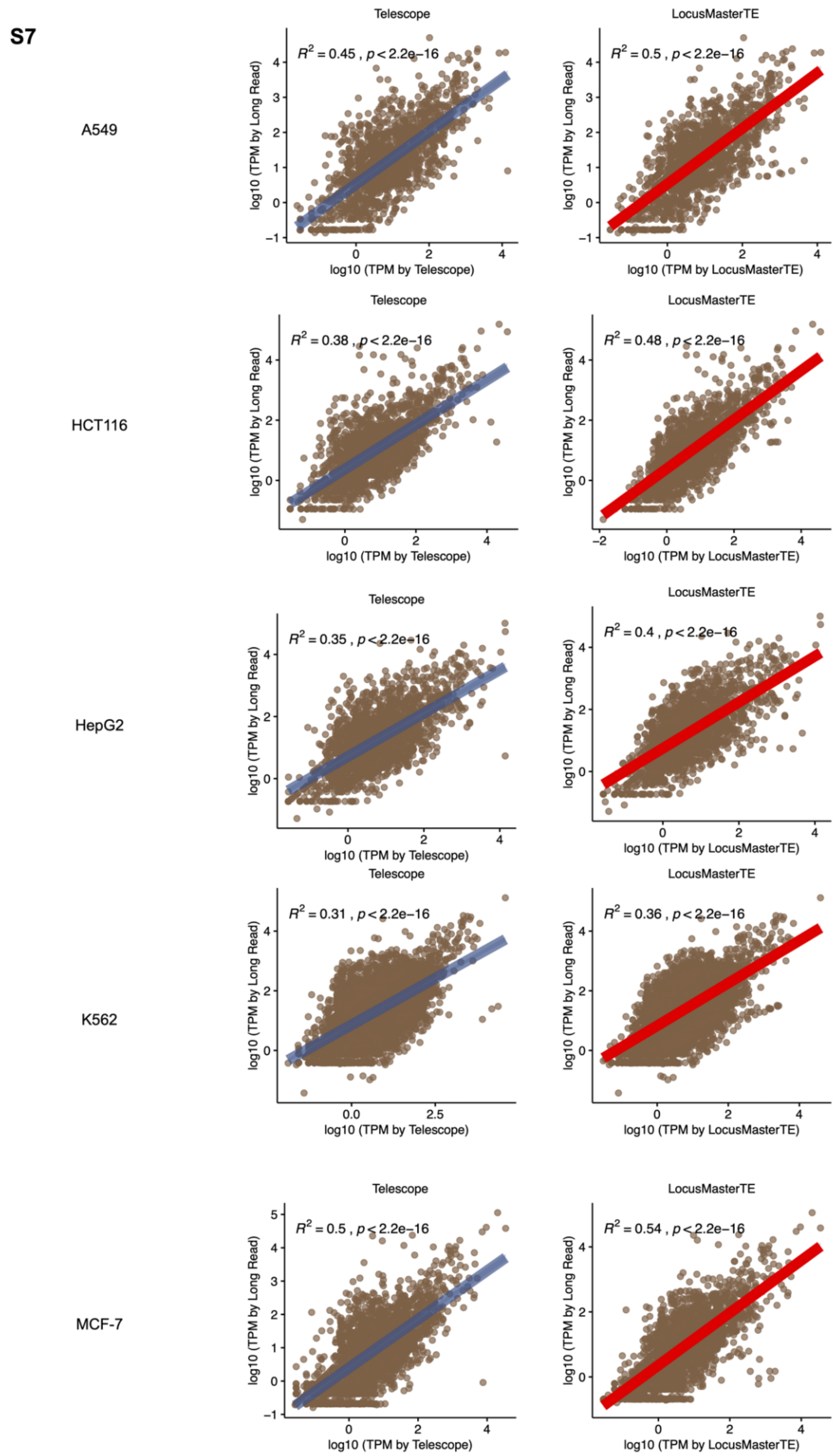

Fig S7: Correlation plot between long-read and short-read for SG-Nex

Series of Spearman correlation plots between long-read and short-read for 5 SG-NEx cell lines. Telescope and LocusMasterTE quantify TEs in 5 cell line samples. Generated TE matrices are compared with the long-read result. Brown: TEs quantified higher in LocusMasterTE (TEs measured highly by LocusMasterTE). Blue: TEs are quantified higher in Telescope (TEs are measured highly by Telescope).

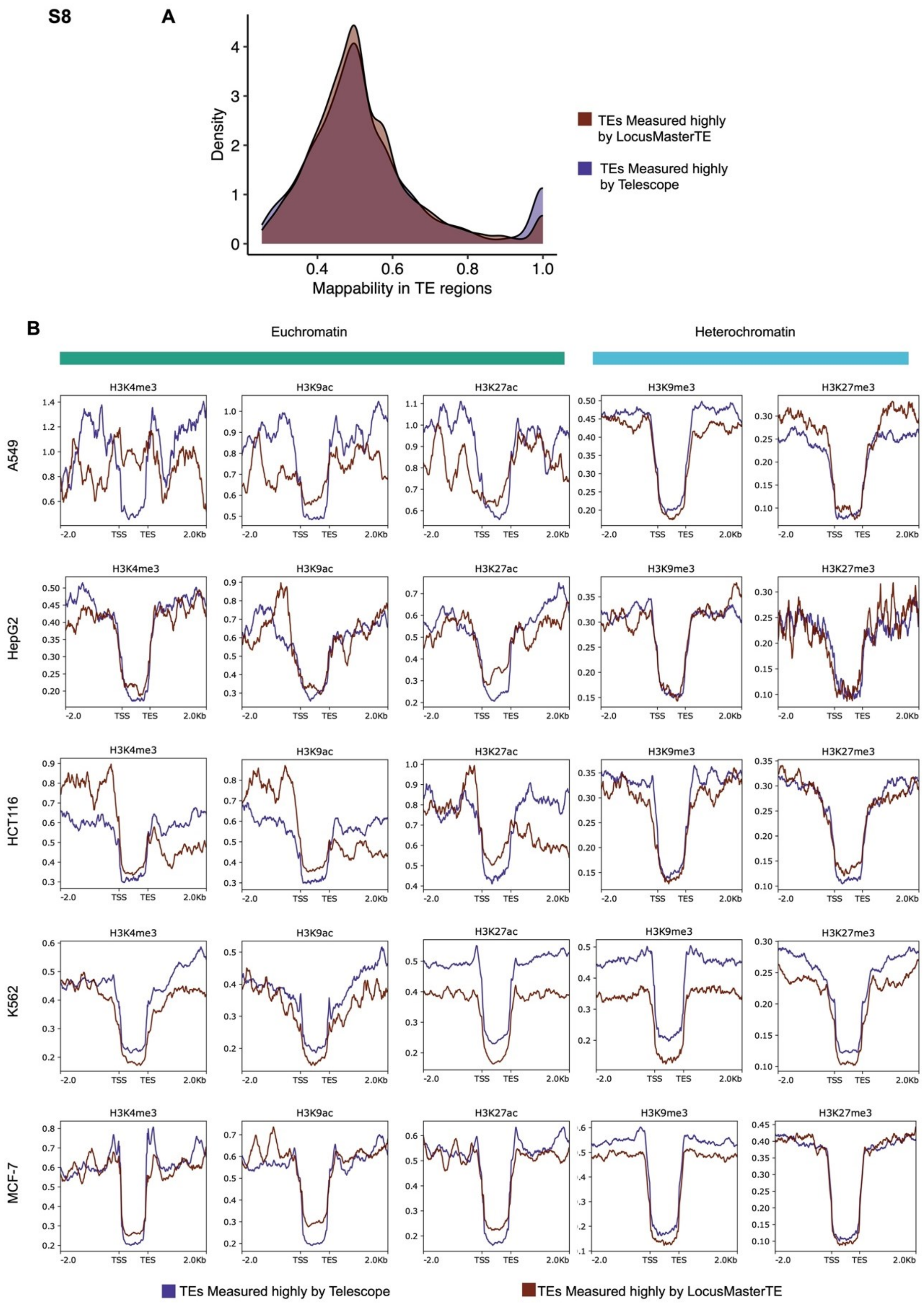

Fig S8: Association with histone marks in SG-NEx dataset.

(A) Distribution graph of average mappability in two groups of TE regions. Mappability > 0.25 was selected. Brown: TEs quantified higher in LocusMasterTE (TEs measured highly by LocusMasterTE). Blue: TEs are quantified higher in Telescope (TEs are measured highly by Telescope). (B)Three active chromatins coverage of 5 SG-NEx samples for two TE groups: TEs quantified higher in LocusMasterTE (TEs measured highly by LocusMasterTE) and TEs quantified higher in Telescope (TEs measured highly by Telescope). Two repressive chromatins coverage of 5 SG-NEx samples for two TE groups, same as above.
